# Image-guided Raman spectroscopy probe-tracking for tumor margin delineation

**DOI:** 10.1117/1.JBO.26.3.036002

**Published:** 2021-03-13

**Authors:** Conor C. Horgan, Mads S. Bergholt, May Zaw Thin, Anika Nagelkerke, Robert Kennedy, Tammy L. Kalber, Daniel J. Stuckey, Molly M. Stevens

**Affiliations:** a Imperial College London, Department of Materials, London, United Kingdom; b Imperial College London, Department of Bioengineering, London, United Kingdom; c Imperial College London, Institute of Biomedical Engineering, London, United Kingdom; d University College London, Centre for Advanced Biomedical Imaging, London, United Kingdom; e King’s College London, Guy’s and St Thomas’ NHS Foundation Trust, Oral/Head and Neck Pathology Laboratory, London, United Kingdom

**Keywords:** Raman spectroscopy, fluorescence guidance, probe tracking, margin delineation, cancer

## Abstract

**Significance:** Tumor detection and margin delineation are essential for successful tumor resection. However, postsurgical positive margin rates remain high for many cancers. Raman spectroscopy has shown promise as a highly accurate clinical spectroscopic diagnostic modality, but its margin delineation capabilities are severely limited by the need for pointwise application.

**Aim:** We aim to extend Raman spectroscopic diagnostics and develop a multimodal computer vision-based diagnostic system capable of both the detection and identification of suspicious lesions and the precise delineation of disease margins.

**Approach:** We first apply visual tracking of a Raman spectroscopic probe to achieve real-time tumor margin delineation. We then combine this system with protoporphyrin IX fluorescence imaging to achieve fluorescence-guided Raman spectroscopic margin delineation.

**Results:** Our system enables real-time Raman spectroscopic tumor margin delineation for both *ex vivo* human tumor biopsies and an *in vivo* tumor xenograft mouse model. We then further demonstrate that the addition of protoporphyrin IX fluorescence imaging enables fluorescence-guided Raman spectroscopic margin delineation in a tissue phantom model.

**Conclusions:** Our image-guided Raman spectroscopic probe-tracking system enables tumor margin delineation and is compatible with both white light and fluorescence image guidance, demonstrating the potential for our system to be developed toward clinical tumor resection surgeries.

## 1 Introduction

Accurate delineation of tumor margins is essential for improving cancer survival rates, as incomplete tumor resection has been shown to significantly reduce long-term survival rates for a range of cancers.^1^^–^^3^ However, the need for maximal resection needs to be balanced with the goal of healthy tissue preservation in order to minimize patient discomfort and functional impairment. Many groups have thus been researching advanced imaging and spectroscopic techniques for improved tumor visualization and margin delineation.^4^^–^^7^ Fluorescence-guided surgery (FGS) has, for example, been employed with great success for the visualization of high-grade gliomas using fluorophores, enabling improved rates of complete resection and progression-free survival relative to conventional microsurgery.^8^^–^^10^ This technique has, however, been somewhat limited by difficulties in the quantification of fluorescence levels due to varying tissue optical properties and low sensitivities for early stage cancers.^11^^–^^13^

As an alternative, many groups have investigated the application of pointwise optical techniques such as fluorescence spectroscopy,^7^^,^^14^^,^^15^ reflectance spectroscopy,^16^^–^^18^ Raman spectroscopy,^4^^,^^19^^,^^20^ optical coherence tomography,^21^^–^^23^ and confocal endomicroscopy^24^^,^^25^ for cancer detection and diagnosis. These techniques probe the optical or endogenous biomolecular properties of the tissue itself, revealing differences between healthy and diseased tissue that can be used to provide accurate diagnoses. For example, fluorescence spectroscopy has been applied to skin cancer diagnosis^26^^,^^27^ and as a quantitative adjunct to fluorescence imaging of gliomas during brain surgery.^11^^,^^15^ Similarly, reflectance spectroscopy has been used for the detection of cervical precancers *in vivo*^16^ and in combination with fluorescence spectroscopy for the *in vivo* detection of breast, brain, and ovarian cancers.^17^^,^^18^^,^^28^ Raman spectroscopy, in particular, has enabled highly accurate *in vivo* diagnosis of a range of cancers including breast, skin, colon, gastric, and esophageal cancers, exploiting the interaction of light with molecular bonds to identify the chemical species present in a sample.^4^^,^^19^^,^^29^^–^^31^

Although Raman spectroscopy has shown much promise in the accurate diagnosis of cancerous tissues, its application to tumor margin delineation is limited. Due to the infrequency of Raman scattering events, which necessitates relatively long acquisition times (∼1  s per acquisition) to generate sufficient signal, pixel-by-pixel measurement of a surgical field of view (FOV) is unfeasible as this quickly leads to imaging times of multiple hours for specimens as small as $1\;{\mathrm{mm}}^{2}$.^32^^,^^33^ Though there have been attempts to reduce Raman spectroscopic imaging times to enable tumor margin delineation, for example, by combining autofluorescence guidance with selective Raman spectroscopic sampling, such approaches still require 12 to 24 min for imaging.^34^^,^^35^

Instead, *in vivo* clinical diagnostic applications of Raman spectroscopy have relied on the collection of point spectra via handheld fiber-optic probes.^19^^,^^32^ Pointwise application in this manner provides diagnostic information at discrete locations rather than a diagnostic image for a large area, as is the case with fluorescence imaging.^18^ Although this approach can provide highly accurate diagnoses of a given point, tumor margin delineation is unrealistic unless clinicians can visualize and record diagnoses for many points simultaneously.^36^ Importantly, despite these shortcomings, Raman spectroscopy offers a wealth of biochemical information that is complementary to the macroscopic morphological information provided by widefield imaging. Therefore, the development of a system that effectively combines spatially co-registered spectroscopic diagnostic information with widefield imaging could vastly improve the clinical utility of handheld Raman spectroscopic probes for tumor margin delineation.

Here we present a new approach for the acquisition of spatial Raman spectroscopic diagnostic information via computer vision tracking of a handheld spectroscopic probe. Our system enables simultaneous recording of both the position and orientation (pose) of the Raman spectroscopic probe as well as the diagnostic spectroscopic information for each measurement acquisition. Together, the data are overlaid onto imaging of the surgical FOV to provide an augmented reality (AR) display of the FOV. We demonstrate our system is capable of accurate lesion mapping of both *ex vivo* human tumor biopsies and *in vivo* mouse xenograft tumors under white light image guidance, providing comprehensive clinical control over diagnostic parameters to enable system tuning to varied clinical contexts. We further show that our system can be extended to include fluorescence image guidance, resulting in improved margin delineation of fluorescent optical tissue phantoms than fluorescence imaging alone. Our image-guided Raman spectroscopic probe-tracking system thus helps to bridge the gap between point-based spectroscopic diagnoses and imaging information, overcoming the trade-off between diagnostic accuracy and FOV that has thus far limited Raman spectroscopic diagnostic systems.

## 2 Materials and Methods

### 2.1 Fluorescence-Guided Raman Spectroscopic Probe-Tracking System and Characterization

The image-guided Raman spectroscopic probe-tracking system consisted of seven key components; a Raman spectroscopic probe with a 2.1-mm diameter tip (EmVision LLC^37^), a spectrograph (QEPro, OceanOptics), a Raman spectroscopy laser source (785 nm, 600 mW, BandW Tek), a 405-nm LED light source (600 mW, Thorlabs), an 8-megapixel iSight camera (Apple) with a 500-nm longpass filter (Edmund Optics), and a computer (Lenovo Thinkpad T460, Intel Core i5-6200U CPU). System control software was developed in the MATLAB 2017B environment, using the MATLAB graphical user interface development environment, and the MATLAB image processing and computer vision toolboxes. The Raman spectroscopic probe was designed for close-proximity or direct contact Raman spectroscopic measurements (working distance: <0.5  mm, 785-nm tissue penetration depth: ∼1  cm in skin, but tissue-dependent).^38^^,^^39^ Probe tracking accuracy was calculated using a video of probe movement in an *ex vivo* margin delineation setting. Computed coordinate locations for each of the fiducial markers and the probe tip were compared to ground truth coordinates, generated as the mean (n=3) user-identified coordinates for each video frame. Mean computation time (n=3) was determined using native MATLAB functions for the core algorithm processing loop during a typical *ex vivo* margin delineation procedure.

### 2.2 *Ex Vivo* Raman Spectroscopic Margin Delineation of a Tissue Model

*Ex vivo* Raman spectroscopic margin delineation was performed on raw chicken tissue specimens, obtained from a local butcher on the day of experiments, using the image-guided Raman spectroscopic probe-tracking system with a 785-nm laser at 100 mW power output and a 1-s integration time. Here, chicken tissue served as a good model for validation of probe tracking accuracy, with strong visual and spectroscopic lipid contrast enabling ready visual and Raman spectroscopic tissue discrimination. This enabled evaluation of probe tracking accuracy independent of Raman spectroscopic diagnostic accuracy. Twenty-five Raman spectra were collected from chicken muscle tissue and 25 from chicken fat tissue for a single chicken specimen. Spectra were cropped to between 600 and $1800\;{\mathrm{cm}}^{-1}$, background subtracted (Whittaker filter λ=100,000), normalized, and filtered (Savitzky-Golay, first order, frame width = 7) before a partial least squares-discriminant analysis (PLS-DA) classification model of the spectra was developed with a Venetian blinds cross validation using PLS Toolbox (Eigenvector Research, Inc.) within the MATLAB environment. Next, between 22 and 26 spectral acquisitions were obtained from additional chicken specimens and the previously developed PLS-DA model used to delineate the fatty tissue. Algorithm-delineated areas of chicken fat tissue, with safety margin sizes of 0, 1.5, and 3 mm, were compared to the ground-truth area and the sizes of true positive, false negative, and false positive regions determined.

### 2.3 *Ex Vivo* Raman Spectroscopic Margin Delineation of Human Cancer Tissues

Ten human skin squamous cell carcinoma (SCC) tumor biopsy samples and four human skin normal biopsy samples were obtained from the Imperial College Healthcare Tissue Bank (ICHTB) (see Sec. 2.8). *Ex vivo* Raman spectroscopic margin delineation was performed on the human biopsy samples using the image-guided Raman spectroscopic probe-tracking system with a 785-nm laser at 100 mW power output and a 1-s integration time. Raman spectra were acquired from each of four of the SCC tumor biopsy samples and three of the normal biopsy samples cancerous tissue (n=201 spectra), normal (muscle/non-cancerous) tissue (n=64 spectra), and normal (fatty) tissue (n=89 spectra) resulting in n=354 total. Spectra were cropped to between 600 and $1800\;{\mathrm{cm}}^{-1}$, background subtracted (Whittaker filter, λ=100,000), normalized, and filtered (Savitzky-Golay, first order, frame width = 7) before a PLS-DA classification model of the spectra was developed with a Venetian blinds cross validation using PLS Toolbox (Eigenvector Research, Inc.) within the MATLAB environment. The PLS-DA model was then applied prospectively to discriminate between cancerous, normal (muscle/non-cancerous), and normal (fatty) tissue on the remaining SCC tumor biopsy samples with confirmation via adjacent hematoxylin and eosin (H&E) stained sections.

### 2.4 Histology of Human Tissues

10  μm cryosections of human tissue biopsy samples were obtained following water embedding and freezing using a Bright OTF cryostat. Cryosections were subsequently formalin fixed and stained with H&E in triplicate for each sample. Histology sample preparation and staining was performed with the assistance of Lorraine Lawrence at Imperial College London’s Research Histology Facility within the Facility for Imaging by Light Microscopy. Imaging of stained sections was performed using a Zeiss Axio Observer widefield inverted microscope with a 20× objective.

### 2.5 *In Vivo* Raman Spectroscopic Margin Delineation of nu/nu Mouse Tumor Xenograft Model

Two female nu/nu mice (12 weeks old, 25 to 30 g) were subcutaneously injected with $1\times 10^{6}$ cells from a human colorectal carcinoma cell line, SW1222, on their right flank. Using the image-guided Raman spectroscopic probe-tracking system, 80 Raman spectra were collected from control mouse tissue *in vivo* and 80 spectra from the SW1222 xenograft tumors *in vivo* at 12 days after implantation. Spectra were cropped to between 600 and $1800\;{\mathrm{cm}}^{-1}$, background subtracted (Whittaker filter, λ=100,000), normalized, and filtered (Savitzky-Golay, first order, frame width = 7) before a PLS-DA classification model of the spectra was developed with a Venetian blinds cross validation using PLS Toolbox (Eigenvector Research, Inc.) within the MATLAB environment. *In vivo* image-guided Raman spectroscopic margin delineation of a SW1222 xenograft tumor in a third mouse was then performed using the image-guided Raman spectroscopic probe-tracking system with a 785-nm laser at 100 mW power output and a 1-s integration time through prospective application of the previously developed PLS-DA model.

### 2.6 Optical Tissue Phantom Construction for Fluorescence Guidance

MDA-MB-231 cells (ATCC) for optical tissue phantoms were grown at 37°C and 5% ${\mathrm{CO}}_{2}$ in high glucose (4.5  g/L) DMEM GlutaMax (Life Technologies) supplemented with 10% (v/v) fetal bovine serum (FBS), 1× penicillin–streptomycin, 1× non-essential amino acids, and 20 mM pH 7.3 HEPES buffer solution. The cell line was authenticated using STR profiling. Prior to inclusion in optical tissue phantoms, cells were trypsinized, spun down at 300×g for 5 min, washed, and then fixed in 4% (v/v) PFA in PBS for 20 min. Optical tissue phantoms that mimic tissue absorption and scattering were prepared using an adaptation of a previously established protocol,^40^ by combining agarose (Sigma-Aldrich), water, PBS (ThermoFisher), FBS (ThermoFisher), homemade intralipid, and human hemoglobin. Homemade intralipid was made by forming a solution of 20% (v/v) sunflower oil and 1% DSPC (Avanti Polar Lipids) in water. Briefly, 200 mg of agarose was dissolved in a 10 mL 1:1 solution of $H_{2}O:\mathrm{PBS}$ and heated at 100°C under constant stirring until a transparent solution was formed. Solution was then allowed to cool slowly to 50°C. Once the solution reached 50°C, 2.6 mL FBS, 200  μL of homemade intralipid solution, 200  μL of human hemoglobin, and 1 mL of $20\times 10^{6}$ fixed MDA-MB-231 cells/mL were added and stirred through the solution. For fluorescence-guided applications, varying amounts of 500  μM PPIX (Sigma-Aldrich) was also added to create 2, 4, and 20  μM PPIX fluorescent optical tissue phantoms. The solution was then poured into a mould (e.g., cavity cut out of chicken tissue), as required, and allowed to cool to room temperature to set before use.

### 2.7 *Ex Vivo* Fluorescence-Guided Raman Spectroscopic Margin Delineation of Tissue Phantoms

Using the image-guided Raman spectroscopic probe-tracking system, 25 to 30 Raman spectra were collected from raw *ex vivo* chicken muscle tissue, raw *ex vivo* chicken fat tissue, and the 0-, 2, 4, and 20-μM PPIX optical tissue phantoms. Spectra were cropped to between 600 and $1800\;{\mathrm{cm}}^{-1}$, background subtracted (Whittaker filter, λ=100,000), normalized to the area under the curve, and filtered (Savitzky-Golay, first order, frame width = 7) before a PLS-DA classification model was developed with Venetian blinds cross validation using PLS Toolbox (Eigenvector Research, Inc.) within the MATLAB environment. *Ex vivo* fluorescence-guided Raman spectroscopic margin delineation was performed on additional raw chicken tissue specimens with 0, 2, or 4  μM PPIX optical tissue phantom inserts using the fluorescence-guided Raman spectroscopic probe-tracking system with a 785-nm laser at 100 mW and a 1-s integration time and the previously developed PLS-DA model applied prospectively. For each specimen, between 10 and 30 spectral acquisitions were obtained and used to delineate the fluorescent optical tissue phantoms. Algorithm-delineated areas of fluorescent optical tissue phantom, with safety margin sizes of 0, 3.5, and 7 mm, were compared to the ground truth area and the sizes of true positive, false negative, and false positive regions determined.

### 2.8 Ethics Statements

Human samples used in this research project were obtained from the ICHTB. ICHTB is supported by the National Institute for Health Research (NIHR) Biomedical Research Centre based at Imperial College Healthcare NHS Trust and Imperial College London. ICHTB is approved by Wales REC3 to release human material for research (17/WA/0161), and the samples for this project (R19022) were issued from the ICHTB Collection.

All animal studies were approved by the University College London Biological Services Ethical Review Committee and licensed under the UK Home Office regulations and the Guidance for the Operation of Animals (Scientific Procedures) Act 1986 (Home Office, London, United Kingdom) and United Kingdom Coordinating Committee on Cancer Research Guidelines for the Welfare and Use of Animals in Cancer Research.^41^

## 3 Results

### 3.1 Image-Guided Raman Spectroscopic System Development

**Fig. 1:**
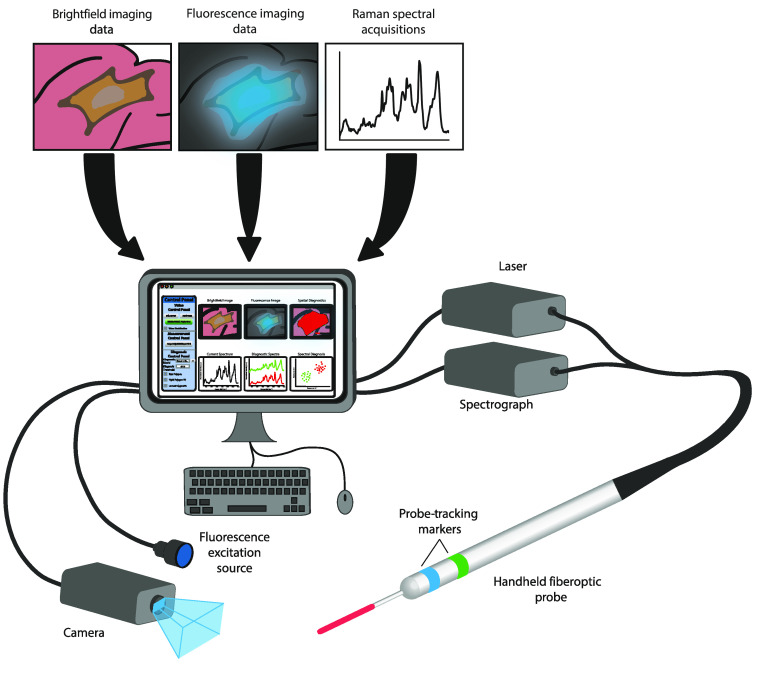
Schematic of the image-guided Raman spectroscopic probe-tracking system comprising a camera for white light/fluorescence imaging, a handheld fiber-optic probe, a laser and spectrograph for Raman spectroscopy, an excitation light source, collection filter optics for fluorescence imaging, and a computer with integrated software for clinical control.

Our image-guided Raman spectroscopic probe-tracking system combines widefield imaging with Raman spectroscopic information and consists of a camera for white light/fluorescence imaging, a handheld fiber-optic probe, a laser and spectrograph for Raman spectroscopy, an excitation light source, collection filter optics for fluorescence imaging, and a computer with integrated software for clinical control (Fig. 1). Clinical application of our system necessitates a probe-tracking algorithm that is robust under varied settings, including different lighting conditions, imaging devices, and spectroscopic probes. We therefore aimed to develop a system that could be readily translated across different environments with minimal technical requirements. To achieve this, we implemented a marker-based visual tracking algorithm that combines visual detection and tracking of colored markers with *a priori* knowledge of probe geometry to determine the position and orientation (pose) of the probe for spatial diagnostics (Fig. S1 in the Supplemental Materials).

In the probe-tracking schema we developed, initial manual identification of the colored, fiducial probe markers in the FOV enables HSV (hue, saturation, value)-based image segmentation (Fig. S2 in the Supplemental Materials), which is then combined with *a priori* knowledge of the probe and marker geometry for ratiometric calculation of the pose of both the spectroscopic probe and the probe tip (point of measurement acquisition) (Fig. S1, I and II in the Supplemental Materials). The HSV color space is used here as it has been shown to be superior to the RGB color space for surgical tool tracking due to its decoupling of the chromaticity and luminance components.^42^^,^^43^ Continuous acquisition (Fig. S1, III in the Supplemental Materials) overlays the tracked location of the probe tip onto the clinical imaging video and records Raman spectral signal in real time. Upon user identification of a region of interest, the system (Fig. S1, IV in the Supplemental Materials) records both the location of the probe tip and a Raman spectral signal for diagnosis. This Raman spectral signal is then diagnosed in real-time through application of a previously developed diagnostic model (e.g., PLS-DA) (Fig. S1, V in the Supplemental Materials) and the diagnoses displayed at the probe tip coordinates are overlaid onto the clinical imaging video. Positive diagnoses are then connected to form a boundary that outlines the lesion margin. Importantly, the algorithm operates independently on each input video frame, accessing any previously stored diagnostic measurements to update margin delineation at each step, to ensure robust performance following temporary occlusion of the spectroscopic probe (Fig. S3 in the Supplemental Materials).

### 3.2 White Light-Guided Spectroscopic Margin Delineation

For *ex vivo* and *in vivo* applications, we implemented our spectroscopic margin delineation algorithm (Fig. S1 in the Supplemental Materials) within a clinician-facing graphical user interface (GUI) that provides functionality for system calibration, diagnostic spectroscopic model development, and margin delineation. The image-guided Raman spectroscopic probe-tracking system control software provides both a raw video input display and an AR display of the surgical FoV, with spatially co-registered spectroscopic diagnostic information overlaid onto the raw video input.

To examine the potential for clinical operation of our system, we first investigated the probe tracking and margin delineation accuracies as well as the computational performance of the system (Fig. S4 in the Supplemental Materials). Validation of our image-guided Raman spectroscopic probe-tracking system was performed using fresh chicken tissue, discriminating between chicken muscle and chicken fat tissues. Here chicken tissue served as a good model for validation of probe tracking accuracy, with strong visual and spectroscopic lipid contrast enabling ready visual and Raman spectroscopic tissue discrimination. This enabled evaluation of probe tracking accuracy independent of Raman spectroscopic diagnostic accuracy. Our probe tracking system achieved a mean probe tip tracking error of 1.07±0.50  mm for a 180-frame video sequence of input size 640×480  pixels with a working distance of 20 cm, in line with existing research and commercial optical tracking systems, which have reported errors of between 0.5 and 4 mm^44^^–^^47^ [Figs. S4(a), S4(c), and S4(d) in the Supplemental Materials]. Similarly, computational performance, measured during a mock spatial diagnostic procedure, yielded processing times of 0.49±0.09  s (0.1-s Raman spectral integration time) for probe tracking and 1.90±0.13  s (1-s Raman spectral integration time) for diagnostic acquisitions [Fig. S4(b) in the Supplemental Materials].

Although probe tracking here is not performed in real time [∼2 to 5 frames per second (fps)] using a laptop, implementation on a more powerful computer together with code parallelization would likely enable real-time performance. Indeed, real-time (>30  fps) surgical tool tracking has been demonstrated using similar visual colored marker-based tracking approaches (∼55  fps)^48^ and more recently with fully convolutional neural network deep learning strategies (∼30  fps).^49^ The most significant impact on computational performance in our system is the integration time required for the acquisition of Raman spectral data (0.1 s for continuous acquisitions during probe movement and 1 s for diagnostic acquisitions). In each case, the integration times applied could likely be significantly reduced (by a factor of up to 10×) as *in vivo* Raman spectroscopic diagnostics has been achieved using integration times as low as 0.1 s.^19^^,^^50^^,^^51^

To further support clinical operation, we implemented several features designed to enable users to tailor spatial spectroscopic diagnostics to a particular patient or lesion (Fig. S5 in the Supplemental Materials). These features include adjustable safety margins, automatic suggested measurement locations, adjustable diagnostic thresholds, and video stabilization. Each of these features is intended to maximize robustness of our system to different clinical settings and maintain clinician diagnostic control.

Using our previously developed PLS-DA model (Fig. S6 in the Supplemental Materials), we evaluated the margin delineation accuracy of our image-guided Raman spectroscopic probe-tracking system for three *ex vivo* chicken tissue specimens. Delineated areas closely resembled the ground-truth areas in each case, as determined via manual image segmentation (Fig. S7 in the Supplemental Materials). Balancing true positive delineated area with false negative and false positive delineated areas, the best results were obtained using a safety margin of 1.5 mm for this particular setup, with a mean delineated true positive area of 86.5%, false negative of 13.5%, and false positive of 21.7% (Fig. S8 in the Supplemental Materials).

**Fig. 2:**
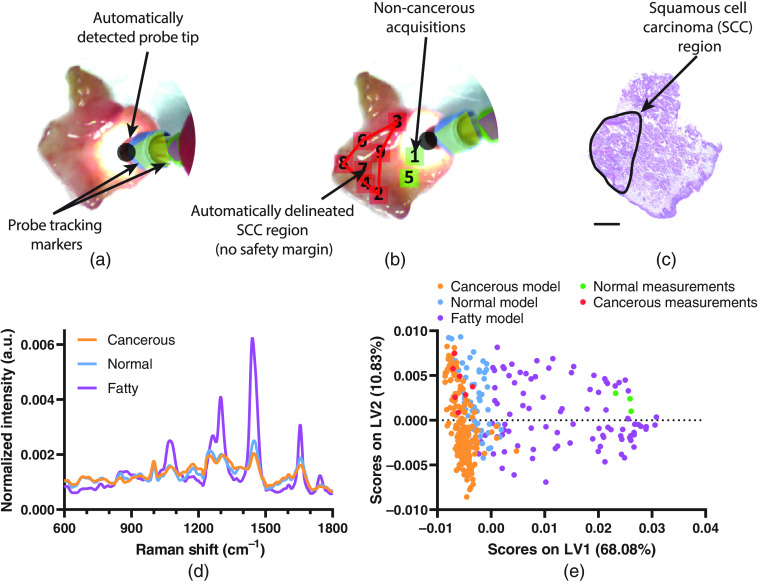
(a) White light image of the bulk SCC biopsy specimen prior to first Raman diagnostic acquisition. (b) White light image of the bulk tumor biopsy specimen with overlaid Raman spectral measurements and tumor margin delineation (red line) (no safety margin shown) where green squares indicate locations of negative (non-cancerous) measurements, red squares indicate locations of positive (cancerous) measurements, and the numbers inside each square indicate order of acquisition. (c) Adjacent H&E stained section of SCC biopsy specimen (scale bar = 2 mm). (d) Mean Raman spectra of cancerous, normal (non-fat), and fatty (normal) tissues (N=3 to 4 tissues, n≥20 spectra). (e) PLS-DA latent variable 1 and 2 (LV1 and LV2) scores for cancerous, normal (non-fat), and fatty (normal) tissues, where PLS-DA latent variables represent spectral features of descending importance that best enable separation of the different tissue classes.

Next, we examined the tissue discrimination capacity of our image-guided Raman spectroscopic probe-tracking system *ex vivo* using a combination of human SCC biopsy samples (n=10 tissues) and human normal (muscle/non-cancerous and fatty) tissue biopsy samples obtained from abdominoplasty procedures (n=4 tissues). Raman spectra were acquired using our image-guided Raman spectroscopic probe-tracking system for cancerous tissue (n=201 spectra), normal (muscle/non-cancerous) tissue (n=64 spectra), and normal (fatty) tissue (n=89 spectra) with a 1-s acquisition time. Together, these spectra were used to develop a PLS-DA model discriminating between cancerous, normal (muscle/non-cancerous), and normal (fatty) tissue with cross-validated accuracies of 92.0%, 90.6%, and 95.7%, respectively (Fig. 2 and Fig. S9 in the Supplemental Materials). Using this model, we were able to effectively delineate a region of cancerous tissue from surrounding normal (muscle/non-cancerous) and normal (fatty) tissue for a human SCC biopsy sample, confirmed histologically using adjacent H&E stained sections (Fig. 2 and Fig. S10 in the Supplemental Materials). The system displays real-time spectroscopic information and automated PLS-DA diagnosis for each acquisition, providing an AR display of the specimen where the locations of negative (non-cancerous) acquisitions are displayed as green squares (with numbers indicating order of acquisition) and the locations of positive (cancerous) acquisitions are displayed as red squares [Fig. 2(b)]. The locations of positive diagnoses are then used to automatically delineate a predicted cancerous region, updated in real time as subsequent diagnostic acquisitions are performed.

### 3.3 White Light-Guided Raman Spectroscopic Margin Delineation in an *In Vivo* Mouse Model

**Fig. 3:**
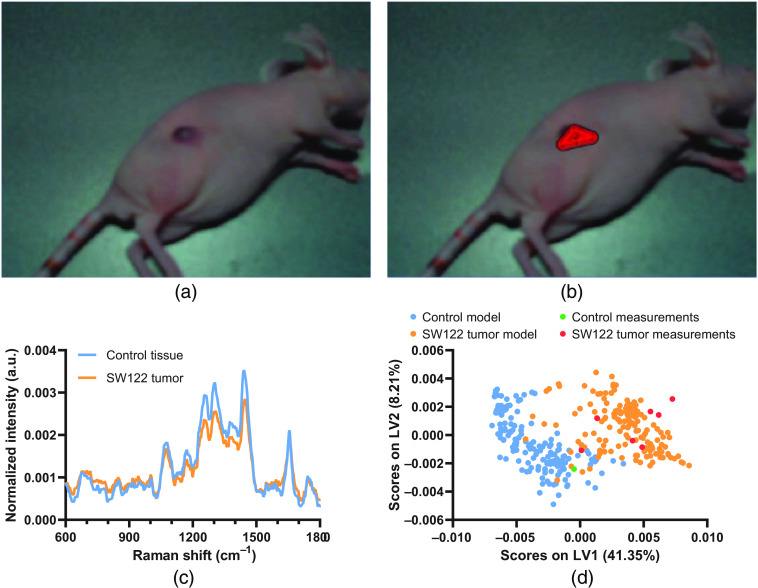
(a), (b) Screenshots from the image-guided Raman spectroscopic probe-tracking system GUI during spatial spectroscopic diagnosis of (a) an SW1222 colorectal xenograft tumor in a nu/nu mouse and (b) with AR Raman margin delineation overlay. (c) Mean Raman spectra of control tissue and SW1222 tumors (N=2, n=80) used for PLS-DA. (d) PLS-DA latent variable 1 and 2 (LV1 and LV2) scores for control tissue and SW1222 tumors.

We next investigated the feasibility of *in vivo* spectroscopic margin delineation in a nu/nu mouse SW1222 colorectal cancer xenograft tumor model (Fig. 3). *In vivo* Raman spectra (n=320) from SW1222 xenograft tumors and control flanks of two mice were collected across multiple timepoints and used to develop a PLS-DA model with a cross-validated accuracy of 96.5% [Fig. 3(d) and Fig. S11 in the Supplemental Materials]. Applying our system together with this PLS-DA model enabled spectroscopic margin delineation of the SW1222 tumor tissue from the surrounding healthy tissue. Crucially this process, in which each spectral acquisition takes ∼2  s, can be performed in as little as 1 to 2 min depending on the complexity of tumor geometry. However, we observed difficulties in performing margin delineation for the 3D SW1222 xenograft tumor due to our use of a single camera, providing only 2D vision. We anticipate that for *in vivo* applications, stereo camera imaging will be required for more accurate probe-tracking in 3D. Although margin delineation accuracy in this case is much harder to calculate than for an *ex vivo* model due to the difficulties in obtaining ground truth data (e.g., co-registered histological imaging), this result points to the clinical potential of our image-guided Raman spectroscopic probe-tracking system for aiding tumor resection procedures in intraoperative settings. While promising, it should further be noted that for such clinical applications, margin delineation accuracy is dependent on both the probe tracking accuracy and the accuracy of the diagnostic model itself, e.g., the PLS-DA model applied here.

### 3.4 Fluorescence-Guided Spectroscopic Margin Delineation: Demonstration in a Tissue Model

Although the *in vivo* and *ex vivo* margin delineation results achieved with our system under white light guidance enabled the precise delineation of tumor margins, they do not address the first essential component for successful tumor resection—the initial detection and identification of suspicious lesions. Indeed, the macroscopic similarity between cancerous and healthy tissues is a key driver behind high-postsurgical positive margin rates for many cancers. Therefore, to extend our system for comprehensive tumor resection assistance, we implemented fluorescence guidance for our Raman spectroscopic margin delineation system.

After examining a range of fluorescent compounds currently employed for clinical and preclinical fluorescence-guidance applications, we selected the photosensitizer compound protoporphyrin IX (PPIX). As we have previously demonstrated,^52^ PPIX displays a low fluorescence background at clinically relevant concentrations under 785 nm Raman excitation (as typically applied in clinical Raman diagnostics) and is currently approved in the US and Europe for fluorescence-guided resection of high-grade gliomas.^12^^,^^53^^–^^55^

In order to quantifiably demonstrate the benefit of fluorescence-guided Raman spectroscopic margin delineation, we developed a series of tissue-mimicking fluorescent optical phantoms containing cellular and serum components as well as varying PPIX concentrations (Fig. S12 in the Supplemental Materials). Raman spectroscopic characterization demonstrated small differences in background fluorescent intensity between the 0-, 2-, and 4-μM PPIX optical tissue phantoms with no obvious loss of Raman spectroscopic information, whereas the 20-μM optical tissue phantom demonstrated considerable fluorescence background and was readily distinguished from the remaining optical tissue phantoms via principal component analysis. Importantly, work quantifying the PPIX levels present in grade IV gliomas following the application of the PPIX precursor, 5-ALA, for FGS has indicated a mean concentration of 5.8  μM^56^ and we have previously demonstrated the possibility of performing Raman spectroscopic diagnostics *in vivo* on PPIX-containing tissues.^52^

**Fig. 4:**
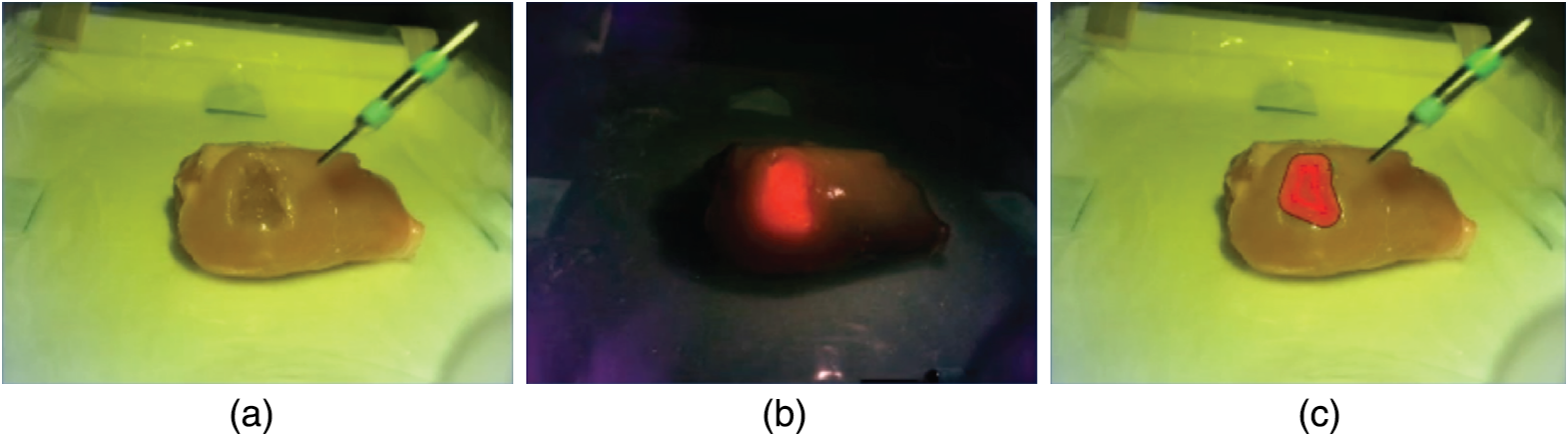
(a)–(c) Screenshots of the image-guided Raman spectroscopic probe-tracking system during diagnosis and margin delineation of a 4-μM PPIX optical tissue phantom inserted into *ex vivo* chicken muscle tissue: (a) white light image; (b) fluorescence image; and (c) AR overlay of Raman margin delineation onto white light image.

We next developed a PLS-DA model to discriminate chicken muscle tissue from a non-fluorescent (0  μM PPIX) optical tissue phantom (100% cross-validated accuracy). Note that as previously, the use of chicken tissue and optical tissue phantoms here enables validation of probe-tracking performance independent of Raman spectroscopic diagnostic performance. We then applied this PLS-DA model for fluorescence-guided Raman margin delineation of both fluorescent and non-fluorescent optical tissue phantoms (Fig. S13 in the Supplemental Materials). We were thus able to both detect fluorescent optical tissue phantoms within chicken tissue via fluorescence imaging and perform margin delineation via Raman spectroscopy (Fig. 4). Although Raman spectral discrimination of the optical tissue phantoms and chicken tissue is trivial due to the significant spectral differences between the two materials, this experimental setup enabled a quantitative comparison of the margin delineation accuracy of our image-guided Raman spectroscopic probe-tracking system and fluorescence imaging alone for a series of 0, 2, and 4  μM PPIX optical tissue phantoms (Fig. S14 in the Supplemental Materials). Although Raman spectroscopic margin delineation accuracy remained constant across the three PPIX concentrations, fluorescence imaging margin delineation was much more dependent on the PPIX concentration, with fluorescence imaging unable to delineate the non-fluorescent optical tissue phantom (0  μM PPIX) (Fig. S15 in the Supplemental Materials). When excluding the 0-μM PPIX optical tissue phantom data results, our image-guided Raman spectroscopic probe-tracking system performs as well as fluorescence imaging alone, with no statistically significant difference in the true positive, false negative, or false positive areas delineated by the two techniques (Student’s t-test, n=6) [Fig. S15(c) in the Supplemental Materials]. However, our image-guided Raman spectroscopic probe-tracking system significantly outperformed fluorescence imaging alone for the 0-μM PPIX optical tissue phantom, with important implications for clinical applications to non-fluorescing tumor regions.

**Fig. 5:**
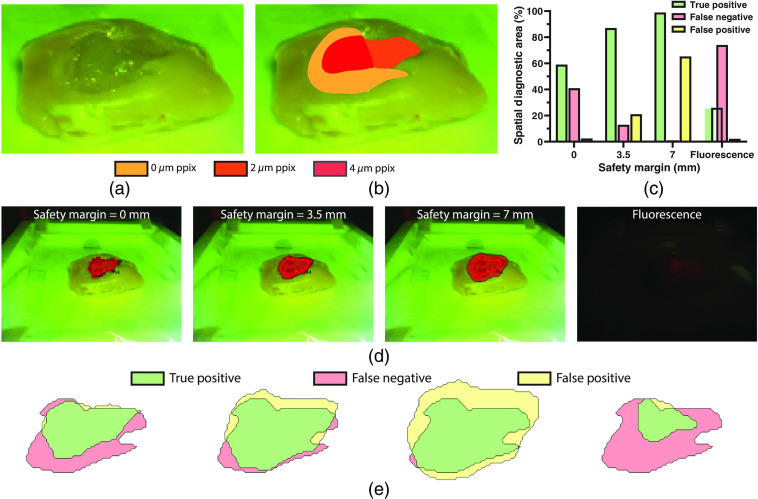
(a) Photograph of the tumor mimicking fluorescent optical tissue phantom with regions of varying PPIX concentration. (b) Photograph of the tumor mimicking fluorescent optical tissue phantom with overlaid map of PPIX concentration regions. (c) Margin delineation accuracy for the tumor mimicking fluorescent optical tissue phantom via fluorescence-guided Raman spectroscopic margin delineation with safety margins of 0, 3.5, and 7 mm, and via fluorescence imaging. (d) Margin delineation of tumor mimicking optical tissue phantom using fluorescence-guided Raman spectroscopic margin delineation (with safety margins of 0, 3.5, and 7 mm) and fluorescence imaging. (e) Corresponding true positive, false negative, and false positive areas for fluorescence-guided Raman spectroscopic margin delineation (with safety margins of 0, 3.5, and 7 mm) and fluorescence imaging. Note that safety margins for the probe-tracking system are dependent on both the probe tracking accuracy and the diagnostic model accuracy.

Next, to better assess the potential of our fluorescence-guided Raman spectroscopic probe-tracking system for tumor margin delineation, we created a composite optical tissue phantom with regions of varied fluorescence intensity (PPIX concentration) (Fig. 5). Studies of FGS for high-grade gliomas have documented varying fluorescence intensity across tumors, particularly in necrotic cores, regions of occluded tissue, and, most importantly, at the tumor margins.^12^^,^^57^ Here margin delineation by our fluorescence-guided Raman spectroscopic probe-tracking system vastly outperformed fluorescence imaging alone (87% true positive diagnostic area with a safety margin of 3.5 mm versus 26% for fluorescence imaging), with our system detecting the optical tissue phantom independent of PPIX concentration. In contrast, fluorescence imaging was only able to detect the 4-μM PPIX region, due to the occlusion of the 2-μM PPIX region under a thin layer (∼1  mm) of 0  μM PPIX optical tissue phantom.

This result thus demonstrates the complementarity of fluorescence imaging with Raman spectroscopic diagnostics, whereby fluorescence imaging enables rapid identification of fluorescence positive regions followed by spatial spectroscopic diagnosis for accurate margin delineation independent of variations in fluorescence intensity. As such, by developing diagnostic models that combine both fluorescence imaging information and Raman spectroscopic information, the two modalities could yield more accurate margin delineation in combination than either modality would achieve in isolation. However, an important caveat to note is that margin delineation accuracy via our image-guided Raman spectroscopic probe-tracking system is dependent on both the probe tracking accuracy and the accuracy of the underlying diagnostic model for spectroscopic discrimination between tumor and healthy tissue. Given the significant spectral differences between the optical tissue phantoms and the chicken tissue used for these experiments, the results presented here reflect an idealized case where the underlying diagnostic model has a 100% discrimination accuracy.

## 4 Conclusions and Outlook

High information content optical spectroscopies such as Raman spectroscopy have long held promise for tumor margin delineation. However, the long acquisition times required have thus far restricted intraoperative Raman spectroscopy systems to point-based applications that do not provide clinicians with a visual demarcation of a tumor boundary.^4^^,^^31^^,^^58^ In contrast, fluorescence imaging enables visualization of a tumor’s extent, though this has typically been limited to high-grade tumors that generate sufficient contrast.^59^ Tumor margin delineation is an important clinical challenge as successful surgical treatment of tumors is highly dependent on the extent of tumor resection and postsurgical positive margin rates remain as high as 15% to 60% for a host of cancers.^60^^,^^61^

Our image-guided Raman spectroscopic probe-tracking system is designed to bridge the gap between the high molecular information content of Raman spectroscopy and the visual-spatial information provided by white light/fluorescence imaging. Through tracking the pose of a Raman spectroscopic probe during spectral acquisitions, our system enables an AR display of the surgical FOV, overlaying spatially co-registered spectroscopic diagnoses onto white light/fluorescence video image input for margin delineation. This combination of spatial and spectroscopic data thereby achieves rapid and accurate spectroscopic tumor margin delineation for both *ex vivo* and *in vivo* settings.

Although our approach to tumor margin delineation has potential, several limitations will need to be overcome in order to enable successful clinical application. First, performance should be enhanced to enable real-time operation. Although much of this enhancement could be achieved through improved code parallelization and computing hardware, this is also likely to require the use of a more sensitive Raman spectrometer in order to reduce signal acquisition times. Second, while the system currently provides a 2D AR overlay of spectroscopic and visual information, robust clinical application will likely necessitate the use of multiple cameras for stereo vision.^62^^,^^63^ This would enable improved depth perception for both probe tracking and margin delineation that could better represent complex tumor contours *in vivo*. Importantly, probe tracking accuracy and safety margin determination are both highly dependent on the working distance between the camera and the sample as well as any magnification optics in use. As such, optimization of these parameters is essential for robust clinical implementation. Finally, for fluorescence-guided approaches, care will need to be taken to develop diagnostic models that include data from tumors with varying fluorescence intensities in order to maximize performance.^64^ Although we have demonstrated fluorescence-guided Raman spectroscopy here using only optical tissue phantoms, we have previously reported on the feasibility of Raman spectroscopic diagnosis of PPIX-containing tissues *in vivo* and *ex vivo.*^52^ Combined, these developments would likely prepare our prototype system for more comprehensive preclinical and clinical testing to evaluate its benefits for tumor resection surgeries.

In conclusion, the combination of Raman spectroscopic diagnosis with fluorescence imaging and computer vision probe tracking thus opens exciting opportunities for clinical tumor resection guidance. Given the improved cancer detection sensitivity spectroscopic methods have consistently shown relative to white light/fluorescence imaging modalities, our image-guided Raman spectroscopic probe-tracking system will likely enable more accurate tumor margin delineation and could lead to improved rates of complete resection and thus better patient outcomes.

## Code, Data, and Materials Availability

Raw data is available upon reasonable request from rdm-enquiries@imperial.ac.uk.
