# Supplementary material for: Image-guided Raman spectroscopy probe-tracking for tumor margin delineation
Source: J Biomed Opt. 2021 Mar 13;26(3):036002. doi: 10.1117/1.JBO.26.3.036002 (PMC7960531; doi:10.1117/1.JBO.26.3.036002)
Supplement: Supplementary file 1 [file JBO_026_036002_SD001.pdf]

Image-guided Raman spectroscopy probe-tracking for tumour margin delineation

Conor C. Horgan,[a,b,c] Mads S. Bergholt,[a,b,c,#] May Zaw Thin,[d] Anika Nagelkerke,[a,b,c,†] Robert Kennedy,[e] Tammy L. Kalber,[d] Daniel J. Stuckey,[d] Molly M. Stevens[a,b,c,*]

[a]Department of Materials, Imperial College London, London, UK.
[b]Department of Bioengineering, Imperial College London, London, UK.
[c]Institute of Biomedical Engineering, Imperial College London, London, UK.
[d]Centre for Advanced Biomedical Imaging, University College London, London, UK.
[e]Guy's and St Thomas' NHS Foundation Trust, Oral/Head and Neck Pathology, King's College London, London, UK.
[#]Current address: Centre for Craniofacial and Regenerative Biology, King's College London, London, UK.
[†]Current address: Groningen Research Institute of Pharmacy, Pharmaceutical Analysis, University of Groningen, The Netherlands.

Supplementary Information

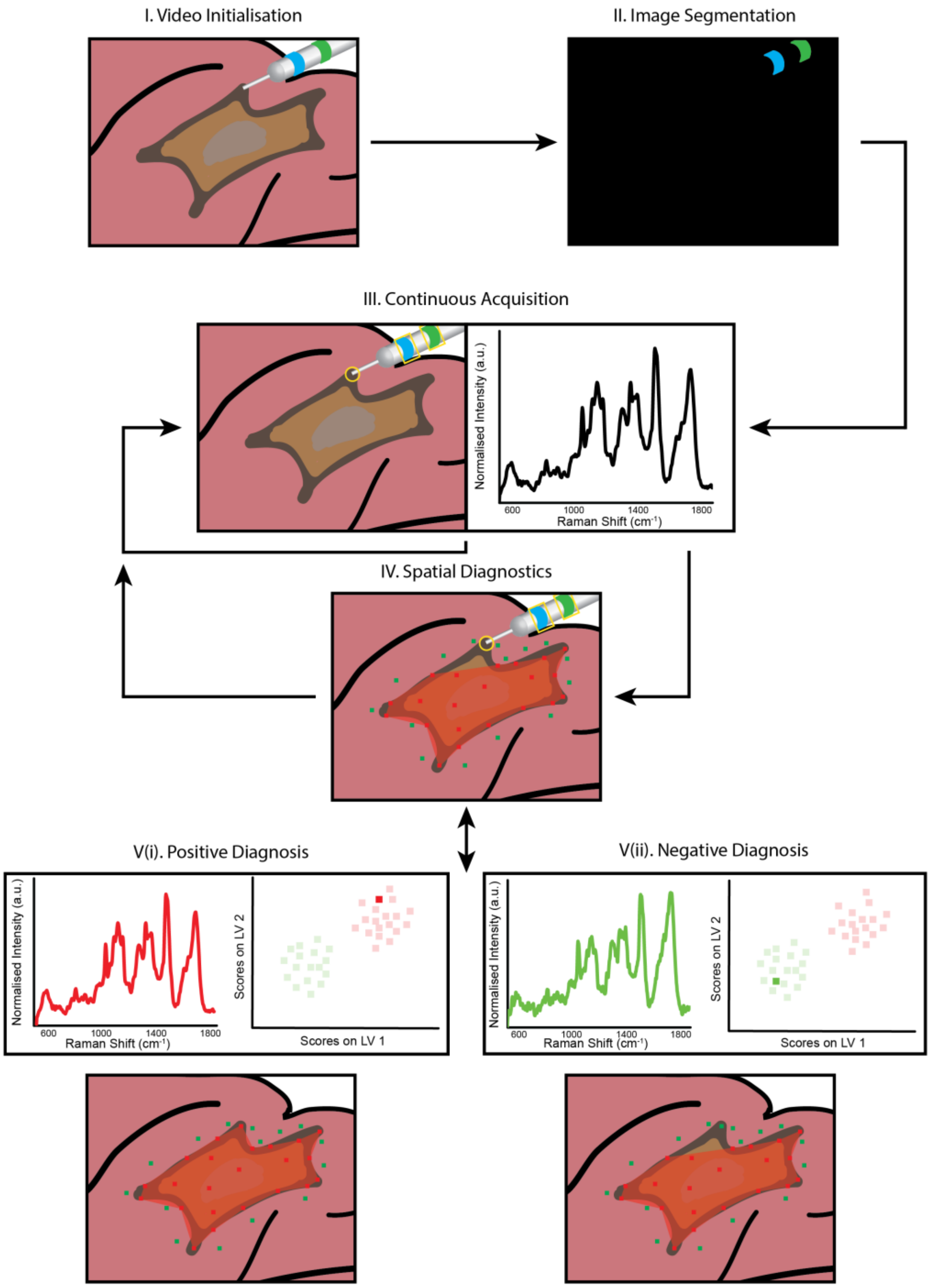

Fig. S1 Schematic of image-guided Raman spectroscopic system software flow; (**I**) Video is initialised with diagnostic probe and interrogation area within FOV, (**II**) User interaction enables coloured-marker-based image segmentation which is combined with probe kinematic information for real-time probe tracking, (**III**) Once selected, the software begins near real-time data acquisition, continually tracking the diagnostic probe and recording spectral diagnostic information until the user starts a diagnostic acquisition, (**IV**) When the user starts a diagnostic acquisition, the coordinates of the probe are recorded and a detailed spectral signal acquired. This spectral signal is then diagnosed as either positive (**V, i**) or negative (**V, ii**) using a pre-developed spectroscopic diagnostic model. The diagnosis is then overlaid onto the imaging information at the coordinates where the measurement was acquired. Positive diagnosis coordinates are connected to form a boundary that delineates the tumour margin.

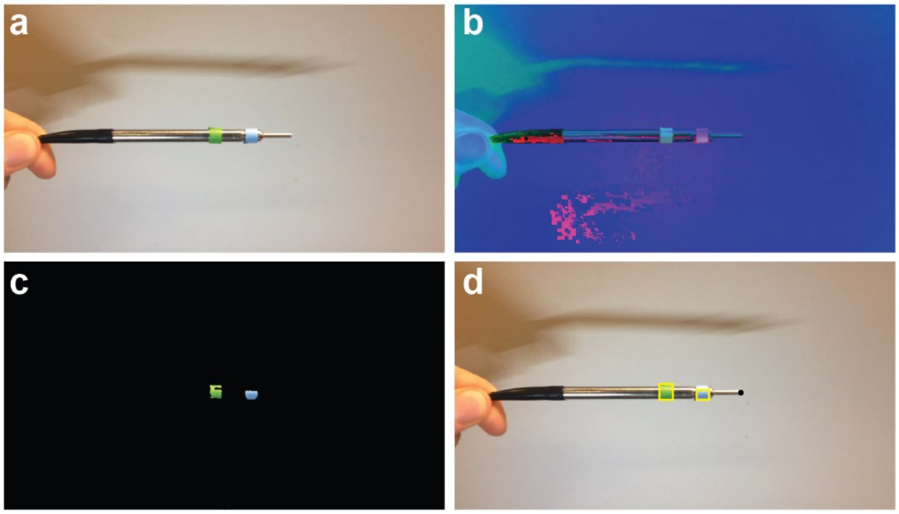

Fig. S2 (**a-d**) Image processing for coloured marker-based tracking takes (**a**) an input image frame and converts it from an RGB image to (**b**) an HSV image before (**c**) HSV-based image segmentation for identification and isolation of the coloured fiducial markers, which (**d**) is then combined with a priori knowledge of the probe geometry to calculate the pose of the spectroscopic probe and the location of the probe tip (black circle).

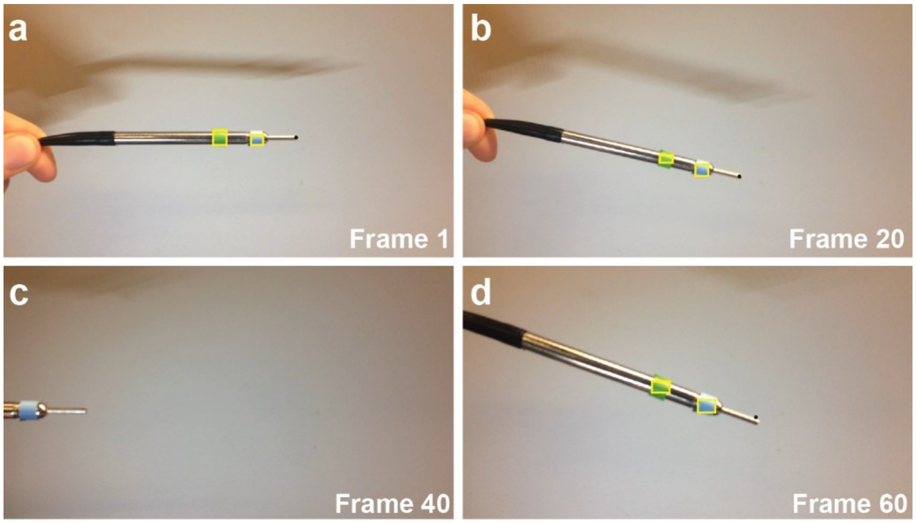

Fig. S3 (**a-d**) Sequential coloured marker-based tracking video frames of the spectroscopic probe during a video sequence, with identified fiducial markers and probe tip, demonstrating robust tracking performance following partial occlusion of the spectroscopic probe.

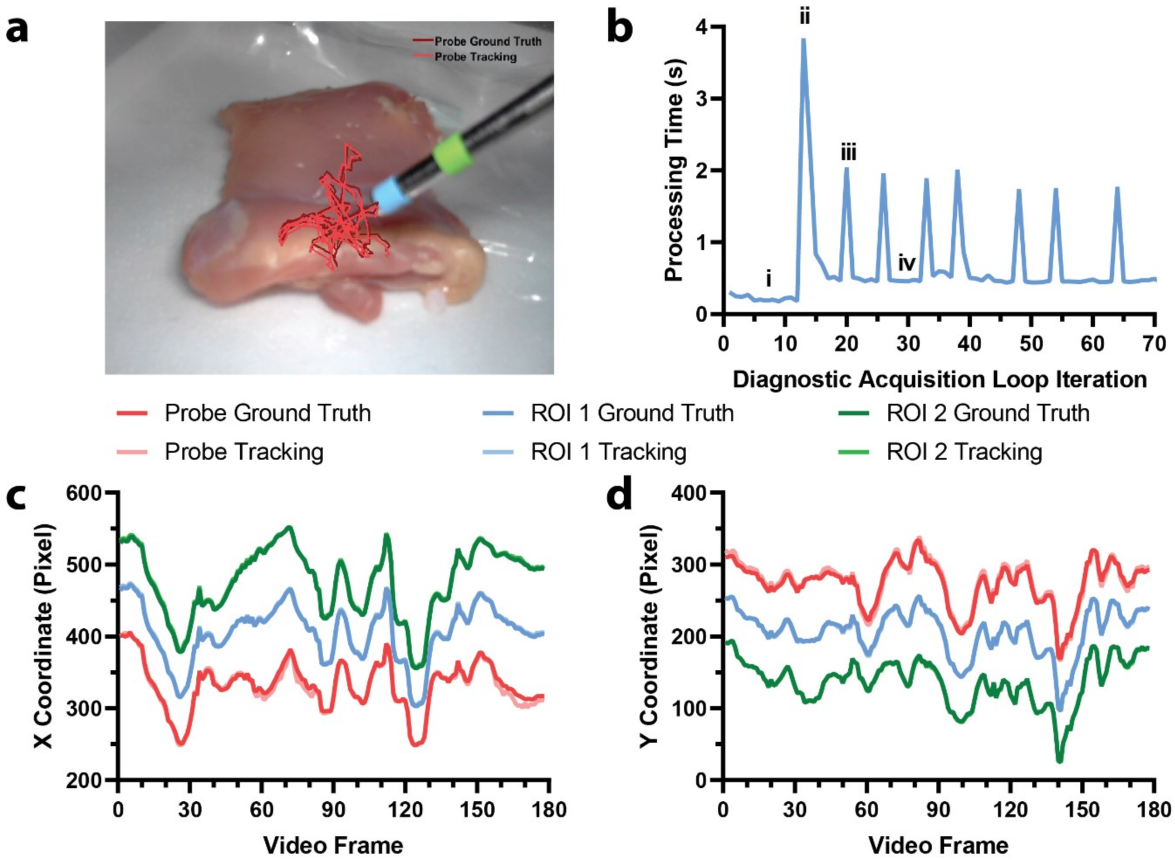

Fig. S4 (**a**) Video frame of mock Raman spectroscopic margin delineation video sequence of fresh chicken tissue used for characterisation of probe tracking error with overlaid ground truth and algorithm-determined probe-tip motion for entire video sequence. (**b**) Analysis of the processing time for the core spectroscopic margin delineation algorithm during (**i**) baseline loop iteration with 0.1 second integration time (prior to spectroscopic diagnostic acquisitions), (**ii**) initial spectroscopic diagnostic acquisition with 1 second integration time, (**iii**) subsequent spectroscopic diagnostic acquisitions with 1 second integration time, and (**iv**) baseline loop iterations with 0.1 second integration time (between spectroscopic diagnostic acquisitions) (n = 3). (**c-d**) Tracking error for probe tip and fiducial markers in (**c**) X and (**d**) Y for the mock spectroscopic margin delineation video sequence.

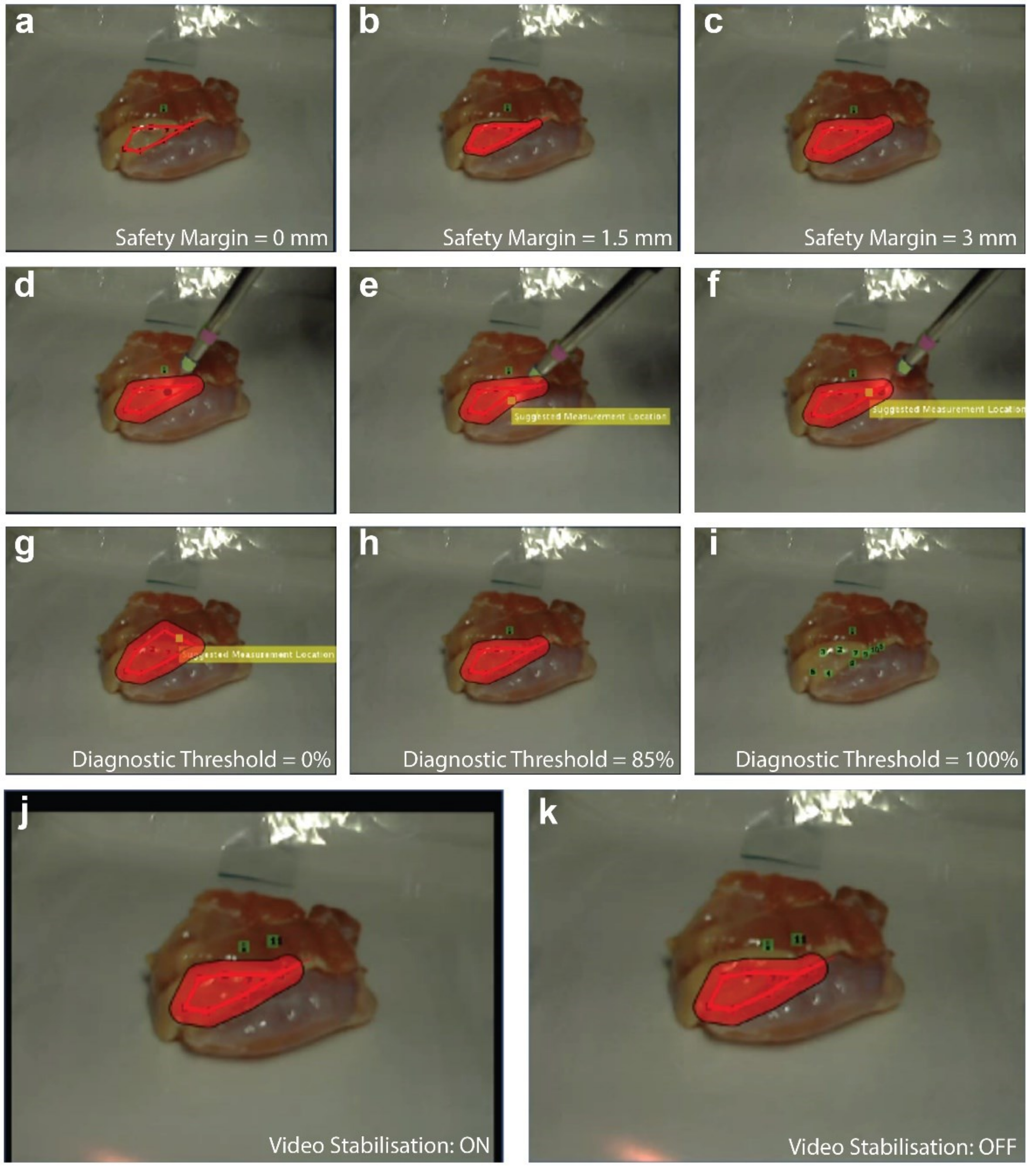

Fig. S5 (**a-c**) Video frames from the image-guided Raman spectroscopic system showing the delineated region of chicken fat tissue with safety margins value of (**a**) 0, (**b**) 1.5 mm, and (**c**) 3 mm. (**d-f**) Sequential video frames from the image-guided Raman spectroscopic system indicating iterative suggested measurement locations (yellow squares) dependent on the spatial distribution of existing positive diagnostic acquisitions. (**g-i**) Video frames from the image-guided Raman spectroscopic system showing the delineated region of chicken fat tissue with diagnostic thresholds of (**g**) 0%, (**h**) 85%, and (**i**) 100% where green squares indicate locations of negative (non-cancerous) acquisitions and red squares indicate positive (cancerous) acquisitions. (**j-k**) Video frames from the image-guided Raman spectroscopic system showing the resulting AR display of spatial spectroscopic diagnostic coordinates when video stabilisation is (**j**) enabled and (**k**) disabled.

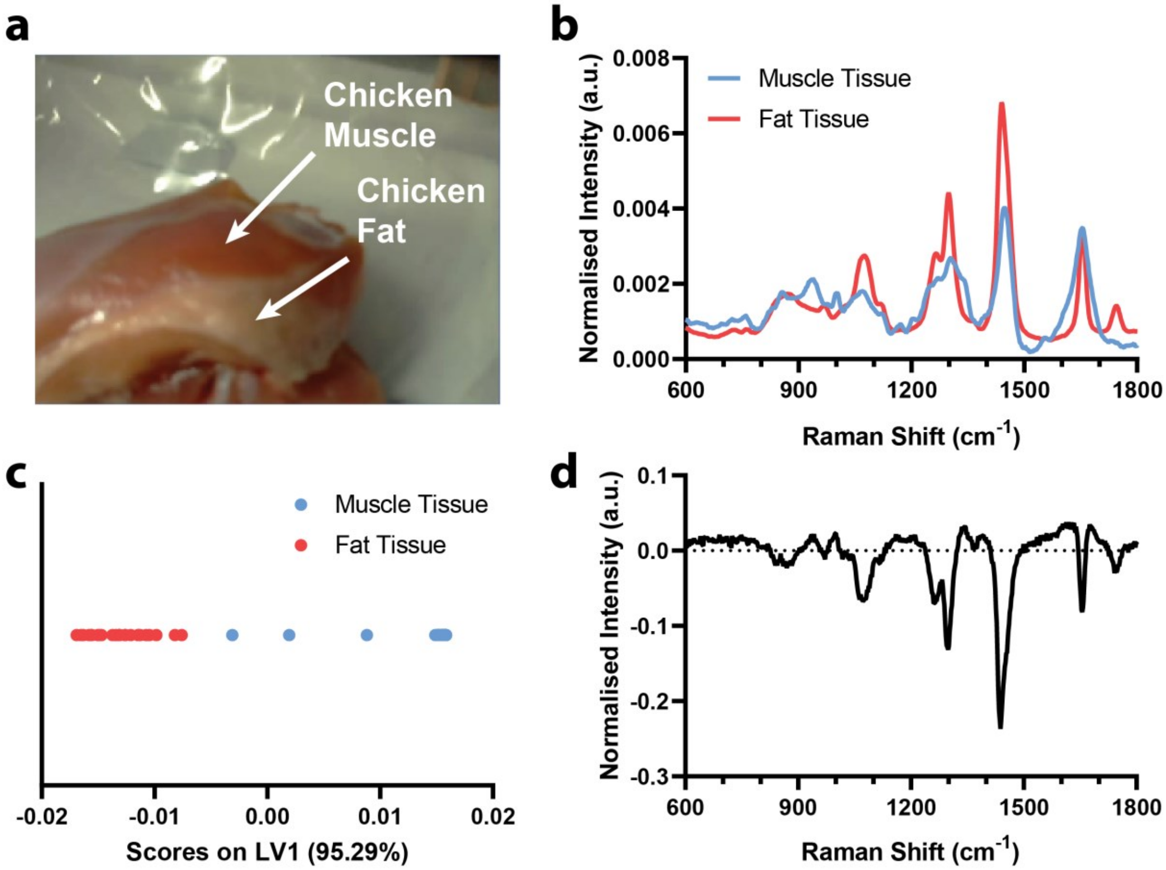

Fig. S6 (**a**) Video frame from the image-guided Raman spectroscopic system showing chicken muscle and chicken fat tissue. (**b**) Mean Raman spectra of chicken muscle tissue and chicken fat tissue (n = 25). (**c**) PLS-DA latent variable 1 (LV1) scores for chicken muscle tissue and chicken fat tissue Raman spectra. (**d**) PLS-DA latent variable 1.

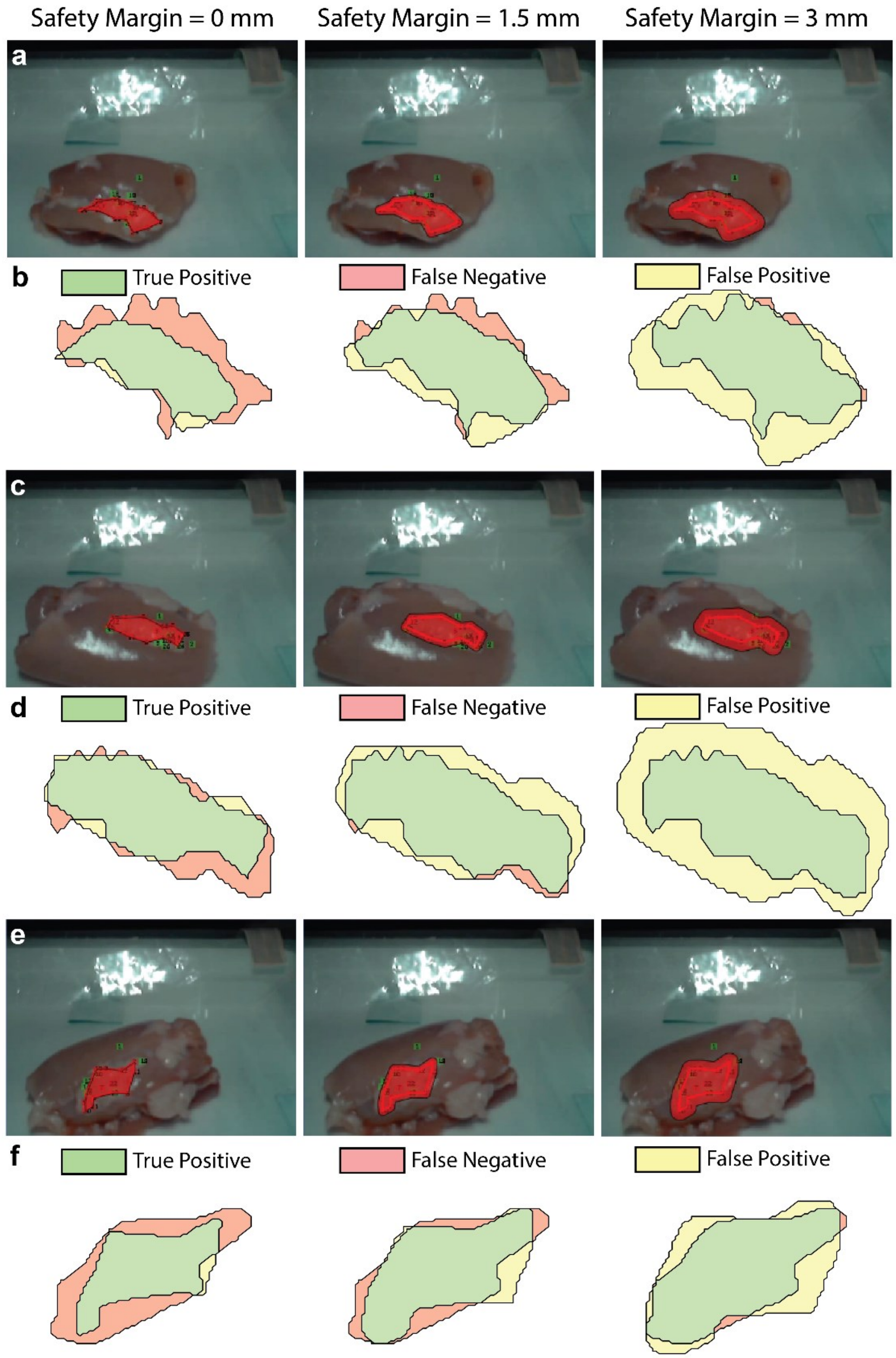

Fig. S7 (**a,c,e**) Video frames from our image-guided Raman spectroscopic system showing the delineated margin of chicken fat tissue following 22-26 diagnostic spectral acquisitions with safety margin sizes of 0, 1.5 mm, and 3 mm for (**a**) specimen 1, (**c**) specimen 2, and (**e**) specimen 3. (**b,d,f**) Corresponding margin delineation accuracies indicating true positive (green), false negative (red), and false positive (yellow) diagnostic regions for (**b**) specimen 1, (**d**) specimen 2, and (**f**) specimen 3.

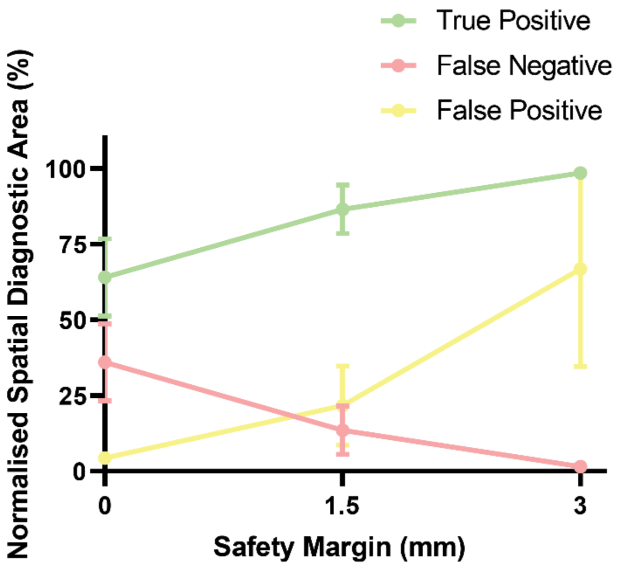

Fig. S8 Mean normalised true positive, false negative, and false positive margin delineated areas for the *ex vivo* chicken tissue specimens.

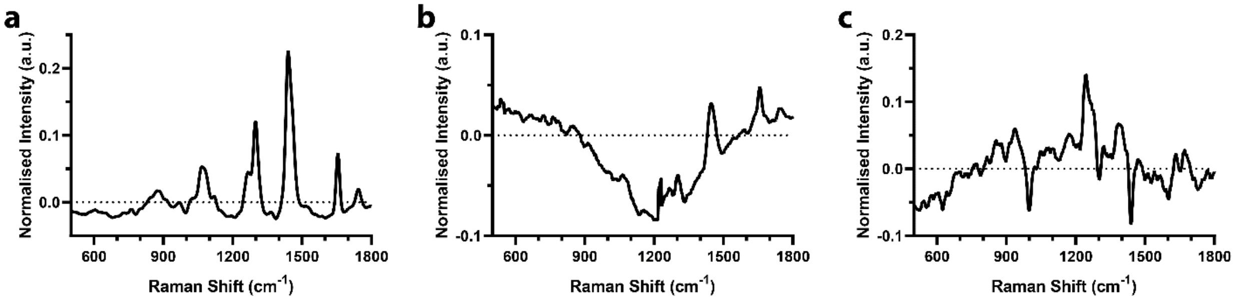

Fig. S9 (a-c) PLS-DA latent variables (**a**) 1, (**b**) 2, and (**c**) 3 for the PLS-DA Raman spectroscopic diagnostic model of human tissue biopsies.

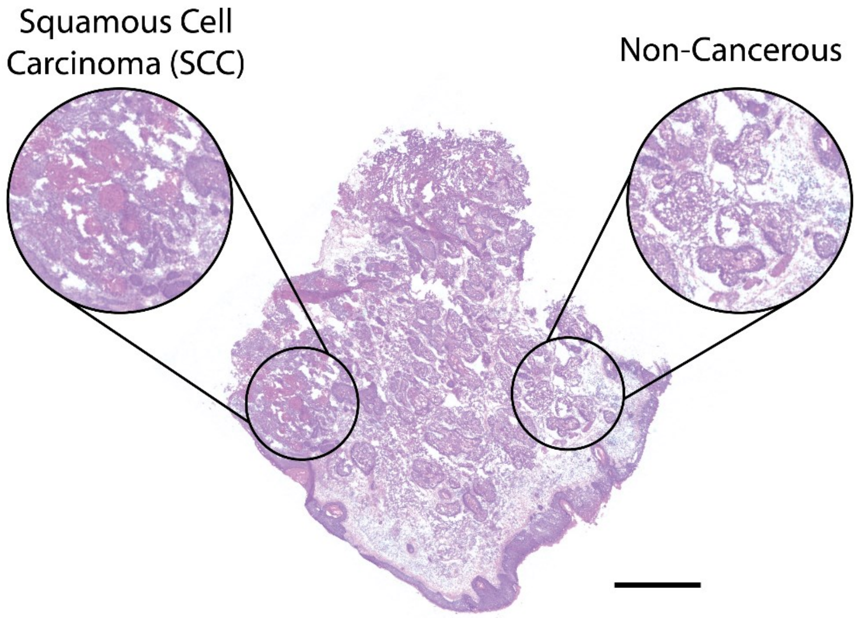

Fig. S10 Magnified H&E stained section of SCC biopsy specimen (scale bar = 2 mm).

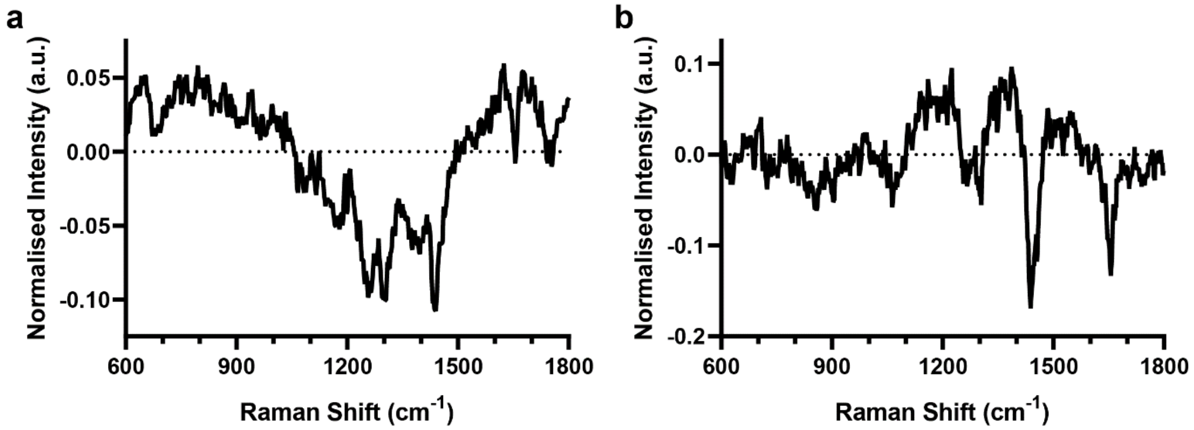

Fig. S11 (a-b) PLS-DA latent variables (**a**) 1 and **(b)** 2 for the PLS-DA Raman spectroscopic diagnostic model of SW1222 colorectal xenograft tumours in *nu/nu* mice.

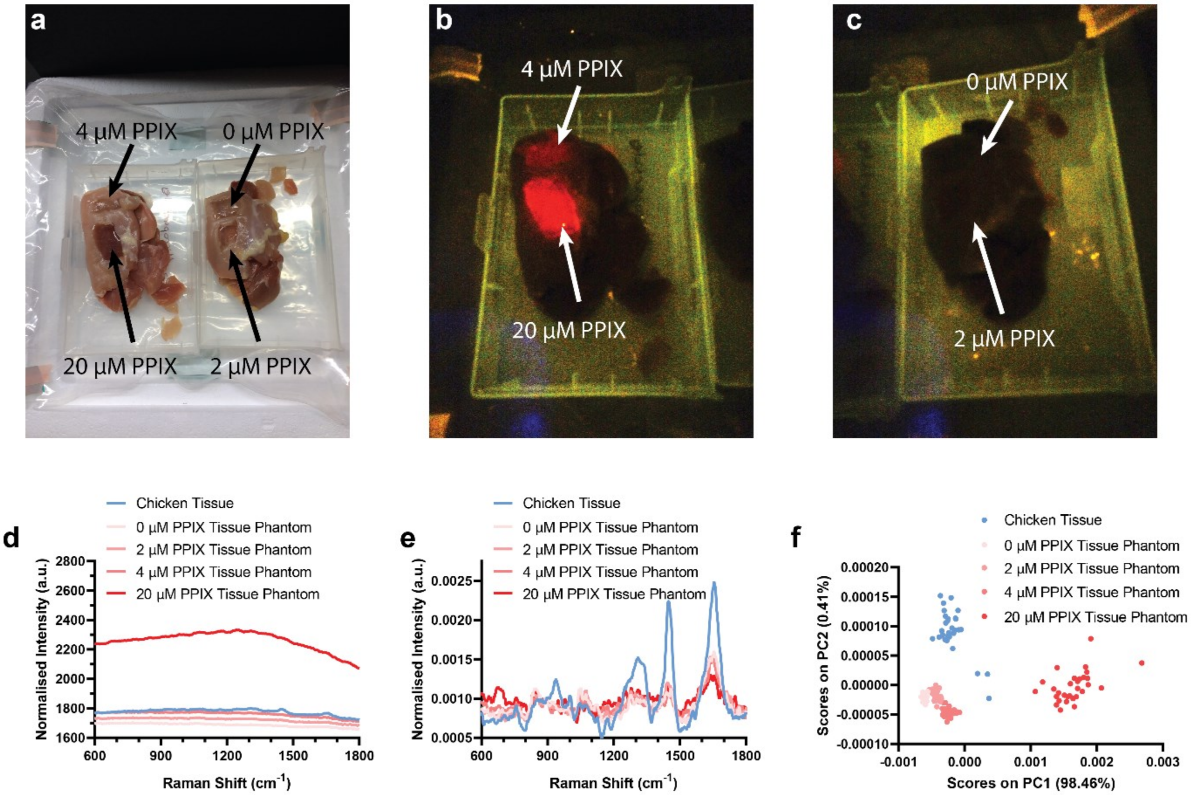

Fig. S12 (**a**) Photographs of 0 μM, 2 μM, 4 μM, and 20 μM PPIX optical tissue phantoms inserted into chicken muscle tissue. (**b-c**) Fluorescence imaging of 0 μM, 2 μM, 4 μM, and 20 μM PPIX optical tissue phantoms in *ex vivo* chicken tissue. (**d**) Raw and (**e**) processed Raman spectra of the PPIX optical tissue phantoms and *ex vivo* chicken muscle tissue (n = 30). (**f**) PCA of the PPIX optical tissue phantoms and *ex vivo* chicken tissue Raman spectra.

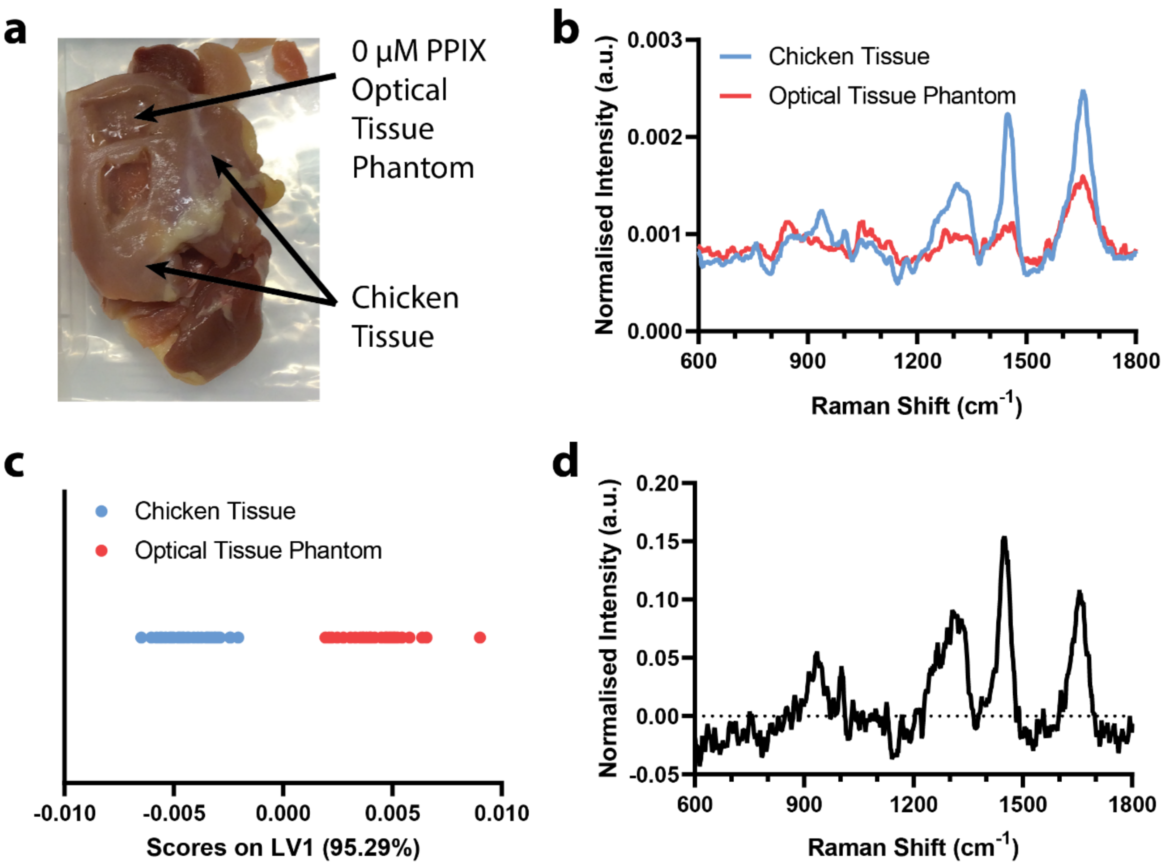

Fig. S13 (**a**) Photograph of the 0 µM PPIX optical tissue phantom inserted into chicken tissue used to generate the PLS-DA model. (**b**) Mean Raman spectra of chicken tissue and optical tissue phantom (n = 30). (**c**) PLS-DA latent variable 1 (LV1) scores for the chicken tissue and the optical tissue phantom Raman spectra. (**d**) PLS-DA latent variable 1.

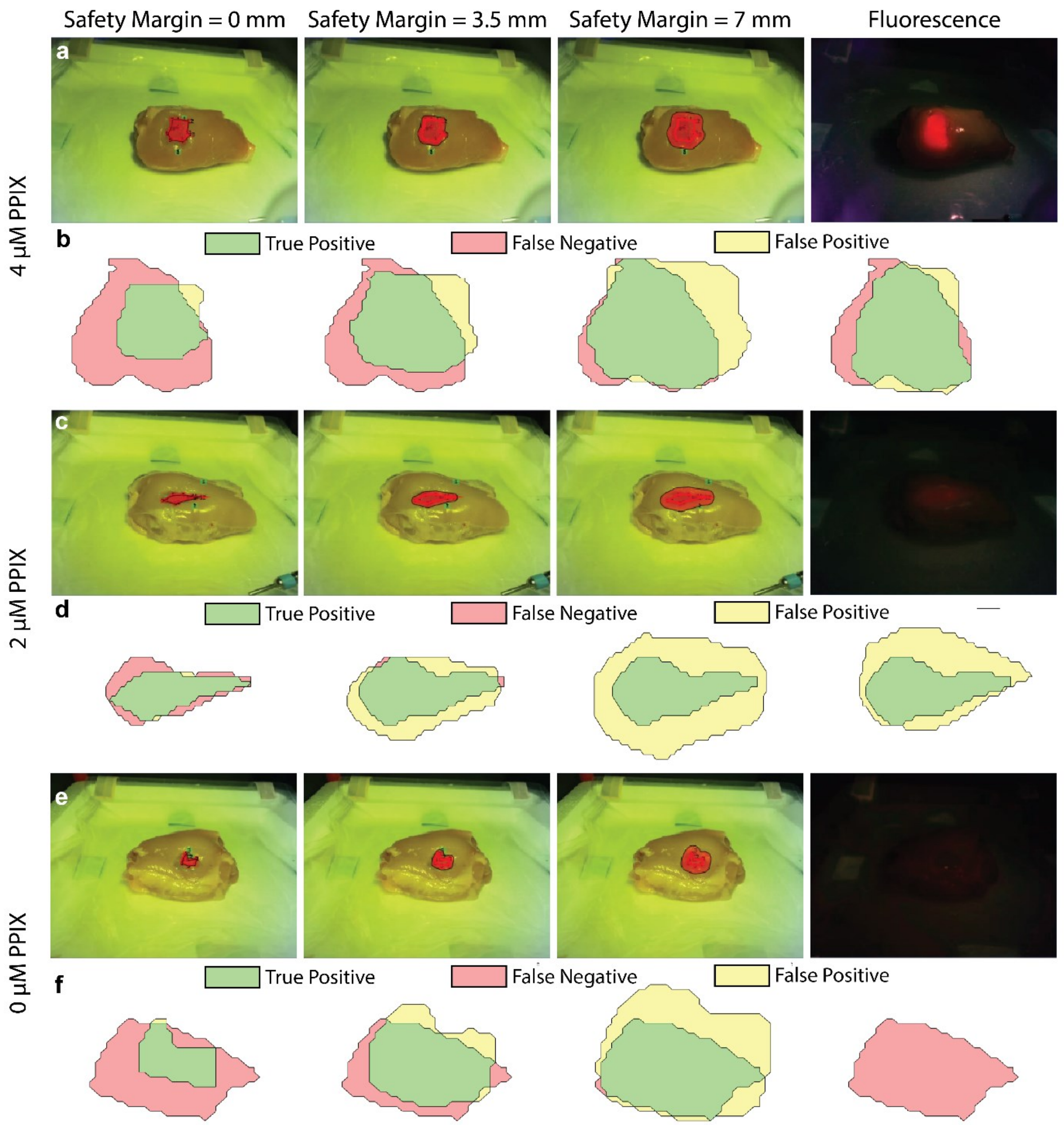

Fig. S14 (**a,c,e**) Exemplar margin delineation of (**a**) 4 μM PPIX, (**c**) 2 μM PPIX, and (**e**) 0 μM PPIX optical tissue phantoms using fluorescence-guided Raman spectroscopic margin delineation (with safety margins of 0, 3.5, and 7 mm) and fluorescence imaging. (**b,d,f**) Corresponding true positive, false negative, and false positive areas for fluorescence-guided Raman spectroscopic margin delineation (with safety margins of 0, 3.5, and 7 mm) and fluorescence imaging.

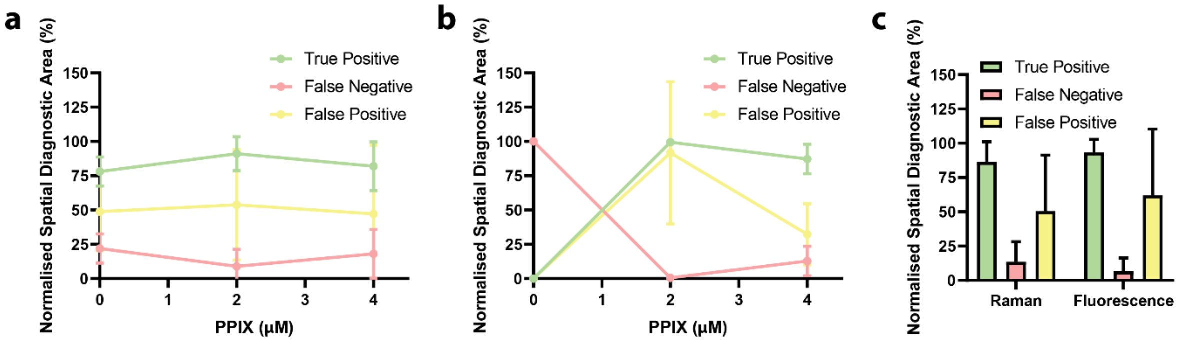

Fig. S15 (a-b) Mean phantom margin delineation accuracy for the 0 µM, 2 µM, and 4 µM PPIX optical tissue phantoms (n = 3) using **(a)** fluorescence-guided Raman spectroscopic margin delineation and **(b)** fluorescence imaging. **(c)** Comparison of the mean margin delineation accuracy of fluorescence-guided Raman spectroscopic margin delineation and fluorescence imaging across the fluorescent optical tissue phantoms (2 µM and 4 µM PPIX).
